# Re-emergence of chikungunya virus in China by 2025: What we know and what to do?

**DOI:** 10.1371/journal.ppat.1013556

**Published:** 2025-10-06

**Authors:** Tong-Yun Wang, Yue Sun, Yan-Dong Tang

**Affiliations:** 1 Department of Cellular and Molecular Medicine, University of California San Diego, La Jolla, California, United States of America; 2 State Key Laboratory for Animal Disease Control and Prevention, Harbin Veterinary Research Institute of Chinese Academy of Agricultural Sciences, Harbin, China; University of Iowa, UNITED STATES OF AMERICA

## Abstract

In July 2025, China witnessed its most significant chikungunya virus (CHIKV) outbreak since 2010. As of August 1, with over 6,000 cases reported in Foshan city, Guangdong Province. Although the clinical manifestations have been relatively mild, the rapid transmission within communities warrants our attention. In this context, we emphasize our current knowledge and the necessary actions to take. Specifically, we identify critical gaps in CHIKV control efforts and assess the effectiveness of current measures. These include vector management strategies, viral genomic surveillance, the deployment of newly approved vaccines, and the development of antiviral agents. Overall, to effectively control the epidemic of CHIKV, we require a comprehensive and multifaceted strategy for its prevention and management.

## 1. What is the chikungunya virus (CHIKV)?

CHIKV is a mosquito-transmitted alphavirus belonging to the family *Togaviridae*, which is characterized by its single-stranded, positive-sense RNA genome and enveloped structure [1–3]. It is primarily transmitted by *Aedes aegypti* and *Aedes albopictus* mosquitoes, which are widely distributed in tropical and subtropical regions [4,5]. The name “chikungunya” originates from the Makonde language, meaning “that which bends” or “to twist,” reflecting the severe joint pain and stooped posture often observed in affected individuals. CHIKV causes an acute febrile illness known as chikungunya fever, which typically presents with symptoms such as fever, headache, rash, myalgia, and severe polyarthralgia. While most cases resolve within a few weeks, a significant proportion of patients experience persistent joint pain and stiffness, which can last for months or even years, leading to long-term disability and socioeconomic burden [1–3]. The virus has three major lineages: West African, East-Central-South African (ECSA), and Asian, with the ECSA lineage further diverging into the Indian Ocean lineage (IOL) [6]. The IOL has shown increased transmissibility due to adaptive mutations in mosquito vectors [7]. Historically, CHIKV was endemic in Africa and Asia, but recent outbreaks have expanded its geographical range. The virus has caused significant public health concerns, particularly in areas with naive human populations and competent mosquito vectors. For instance, the outbreak in Foshan City, Guangdong Province, China, in 2025 represents the most extensive episode of local transmission since 2010. As of August 1, more than 6,000 cases have been reported, the majority of which have been mild in severity [8]. Genomic analysis demonstrated a high degree of homology among these viral strains, all of which are classified within the Central African Clade of the ECSA genotype [9]. Although no severe cases were recorded, the rapid rise in case numbers and the widespread occurrence of community transmission suggest a highly efficient local transmission cycle.

## 2. What factors drive the global spread of CHIKV?

### 2.1. Vectors distribution

Female *Ae. aegypti* and *Ae. albopictus* serve as primary vectors of CHIKV within endemic regions [7]. Therefore, the distribution patterns of both *Aedes* species have become a major driving factor in the global spread of CHIKV [7]. *Ae. aegypti* originated in Africa and was later introduced to the Americas. It is currently predominantly distributed in tropical and subtropical regions, including northern Brazil and Southeast Asia [10]. *Ae. albopictus* was first identified in Asia and subsequently spread to islands in the Pacific and Indian Oceans [11]. Compared to *Ae. aegypti*, *Ae. albopictus* exhibits a greater capacity to survive in cooler climates, which contributes to its broader geographic distribution and increases its potential for disease transmission [12,13]. However, climate change is projected to alter the future geographic distribution of both *Ae. albopictus* and *Ae. aegypti*. In particular, rising global temperatures are expected to drive further range expansion of both mosquito species [14].

### 2.2. International travel and trade

Furthermore, increased international travel and trade, primarily driven by air travel, is contributing to a rise in the frequency and geographic spread of infectious disease epidemics [15]. Human mobility and trade enable rapid connectivity between any two locations worldwide, which has the potential to accelerate the dissemination of emerging and re-emerging infectious diseases, including CHIKV, thereby posing a significant threat to global health security [16].

### 2.3. Virus evolution

Lastly, the adaptation of the virus itself within mosquito vectors constitutes another major driving factor. Since 2000, the virus has expanded its geographic range due to mutations that enhance its ability to infect and be transmitted by *Aedes* mosquitoes, particularly *Ae. albopictus* [17]. The alanine (A)-to-valine (V) substitution at amino acid position 226 of the E1 envelope glycoprotein (designated as the E1-A226V mutation), first identified during the 2005–2006 epidemic on La Réunion Island, is a key mutation that significantly increases the virus’s fitness in *Ae. albopictus* and facilitates its spread into new regions [18]. In 2010, Guangdong Province in China experienced a large-scale CHIKV outbreak involving the E1-A226V mutation [19]. Since then, the virus has continued to evolve, with new variants emerging in South Asia and spreading to Europe and the Americas [7]. The recent resurgence of CHIKV in China and Southeast Asia highlights the urgent need for a robust, evidence-based public health response.

## 3. What challenges do we currently face?

### 3.1. Effective vector control

In fact, alongside CHIKV, other emerging and re-emerging vector-borne diseases (VBDs), such as dengue virus (DENV) and Zika virus (ZIKV), also present significant public health challenges. The World Health Organization (WHO) advocates for an integrated vector management (IVM) strategy as a means to combat the transmission of VBDs [20]. Consequently, controlling mosquito vectors is essential for addressing the full spectrum of VBDs, including CHIKV. IVM represents a comprehensive approach to managing VBDs by optimizing resource utilization, fostering collaboration among health, environmental, and other sectors, and integrating various control strategies, including environmental management, biological control methods, and the judicious application of chemical interventions [20]. IVM promotes sustainable and cost-effective disease control by emphasizing evidence-based decision-making, community engagement, and adaptive management in response to changing environmental and climatic conditions. Despite its recognized importance, the implementation of IVM remains limited in many endemic countries due to challenges such as insufficient infrastructure, financial resources, and human capacity. However, successful IVM programs demonstrate the potential to reduce disease burden, enhance public health outcomes, and support global health goals, including the Sustainable Development Goals. The local government in China has placed considerable emphasis on the recent epidemic, coordinating various functional departments to implement mosquito eradication initiatives. These efforts have produced remarkable outcomes, and the transmission rate of the epidemic is now effectively under control. Therefore, strengthening IVM through policy development, intersectoral coordination, and continuous innovation is critical for effectively combating vector-borne diseases and improving global health security.

### 3.2. Genomic evolution and surveillance gaps

Real-time genomic surveillance is essential for understanding the transmission dynamics and evolutionary patterns of CHIKV [7]. However, a significant gap remains in the availability of full-genome sequences from the 2025 outbreaks in China and Southeast Asia. This limitation may severely hinder the ability to trace the origin of the virus, identify key mutations, and elucidate its transmission pathways. Although there is currently no conclusive evidence suggesting that existing vaccines offer superior protection against specific viral lineages compared to others, it is essential to comprehend the sequence information related to the ongoing epidemic-such as viral lineages, virulence factors, and transmission dynamics-for effective public health interventions. This understanding facilitates the assessment of epidemic risks and the development of targeted prevention and control strategies. For example, the E1-A226V mutation, a defining feature of the IOL, is known to enhance viral fitness in *Ae. albopictus* and was detected in over 90% of subsequent viral sequences from Réunion Island [21]. In the absence of genomic data, it remains uncertain whether the current outbreak is attributable to a novel variant or the re-emergence of previously circulating strains. We strongly recommend that affected countries promptly initiate high-throughput sequencing of clinical samples and adhere to the FAIR (Findable, Accessible, Interoperable, Reusable) principles for data sharing. After the outbreak of the epidemic in Guangdong, the Guangdong Provincial Center for Disease Control and Prevention conducted sequencing on the E1-E2 fragments from the initial batch of cases. The results indicated that the virus belongs to the ECSA-V sublineage, characterized by a consistent presence of E1-226V. Furthermore, it exhibits two distinctive mutations associated with “Indian Ocean-Southeast Asia”: E2-I211T and nsP3-S259P. These findings reveal a close genetic relationship with strains isolated from Sri Lankan tourists in 2023. By analyzing inbound flight data, it can be inferred that the source of introduction is linked to routes connecting South Asia and Southeast Asia. Of note, after the outbreak of the epidemic, the National Microbial Science Data Center at the Institute of Microbiology, Chinese Academy of Sciences, recently developed a Chikungunya Virus Database (CHIKVdb, https://nmdc.cn/gcpathogen/chikv) to support current and future epidemic responses [22]. This database integrates 8,193 global CHIKV nucleic acid sequences (including full-length and fragment sequences) and features a dedicated section for China to facilitate localized prevention and control efforts. These actions will enable researchers and public health authorities to monitor the virus’s evolution, anticipate its spread, and support the development of targeted public health interventions.

### 3.3. Vaccination in areas at risk for CHIKV

Vaccination is the cornerstone of public health [23,24]. In 2023 and 2025, a live attenuated CHIKV vaccine and a virus-like particle vaccine were approved by the U.S. FDA, marking a significant advancement in the fight against CHIKV [25]. These vaccines may provide a promising tool for preventing infection and alleviating the burden of disease [26]. However, their availability remains limited across Asia. While certain countries, such as Thailand, have recommended vaccination for travelers to endemic regions, a comprehensive national deployment strategy is still under development in many Asian nations [27]. In areas with high transmission rates, such as Foshan, mass vaccination campaigns could be implemented. Healthcare workers and frequent travelers should also be considered for vaccination to maintain essential services and prevent cross-border transmission.

### 3.4. Lack of antiviral agents

**Fig 1:**
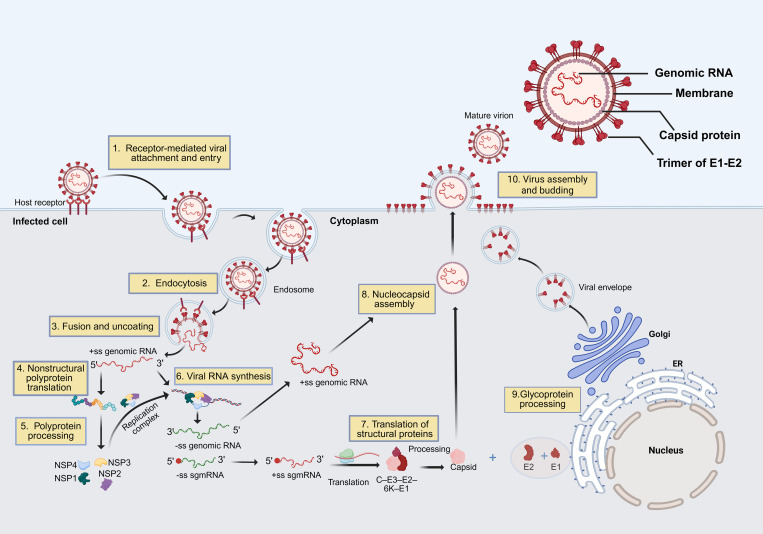
The chikungunya virus (CHIKV) replication cycle includes several steps, each offering potential targets for antiviral design. (Step 1) The E2 glycoprotein of CHIKV binds to cellular attachment factors (e.g., heparan sulfate and phosphatidylserine receptors) and the receptor MXRA8, facilitating viral attachment. (Step 2) CHIKV enters host cells via clathrin-mediated endocytosis. (Step 3) In the endosome, an acidic environment induces a conformational change in the E1 glycoprotein, leading to fusion of the viral envelope with the endosomal membrane and release of viral genomic RNA into the cytoplasm. (Step 4) The genomic RNA is positive-sense single-stranded RNA (+ssRNA), functioning as messenger RNA (mRNA). Translation begins at the 5′ end, producing a large nonstructural polyprotein. (Step 5) This polyprotein is processed by viral proteases, mainly nsP2, into individual nonstructural proteins (nsP1-nsP4). (Step 6) Nonstructural proteins assemble on intracellular membranes to form viral replication complexes (RCs), where +ssRNA serves as a template for synthesizing negative-sense RNA intermediate (−ssRNA). This intermediate is then used to produce full-length +ssRNA and subgenomic +ssRNA. (Step 7) Subgenomic RNA encodes a polyprotein precursor containing structural proteins Capsid–E3–E2–6K–E1, which are processed by host and viral proteases. (Step 8) The capsid protein undergoes autocatalytic cleavage to release itself from the polyprotein and binds full-length +ssRNA to form nucleocapsids. (Step 9) Remaining structural proteins E1 and E2 are co-translationally translocated into the endoplasmic reticulum (ER), where they undergo glycosylation and heterodimer formation before being transported through ER-Golgi intermediate compartment (ERGIC), contributing to viral envelope formation. (Step 10) The nucleocapsid moves to the plasma membrane or membranes derived from the endoplasmic reticulum, where E1 and E2 glycoproteins are incorporated. Budding occurs at these locations, leading to the secretion of virions from the infected cell. This figure created in BioRender. Zhu, Z. (2025) https://BioRender.com/9slnvj3.

Currently, there are no approved antiviral drugs available. This deficiency is particularly critical for the substantial patient population suffering from debilitating, long-term sequelae, most notably a chronic, often erosive, polyarthralgia [28]. The pathogenesis of this condition is multifactorial, underpinned by a complex interplay between the persistence of viral RNA and antigens within synovial tissues, such as fibroblasts and macrophages, and a dysregulated, pro-inflammatory host immune response characterized by elevated levels of cytokines including IL-6, TNF-α, and IL-17 [29,30]. Consequently, addressing this unmet clinical need necessitates an intensified research focus on developing effective therapeutics. The replication cycle of cells infected with CHIKV is illustrated in Fig 1. This provides a foundation for the rational design of therapeutic agents aimed at various stages of viral replication. Therefore, a deeper mechanistic understanding of the entire CHIKV replication cycle is paramount for this endeavor. Key vulnerabilities lie within the viral replicative machinery, for example, the nonstructural protein 2 (nsP2) protease representing a particularly tractable target due to its essential role in polyprotein processing and the availability of its crystal structure for inhibitor design [31]. Furthermore, structure-based drug design may accelerate the development of clinically viable drugs for combating CHIKV.

## 4. Conclusions

The re-emergence of CHIKV in East Asia and Southeast Asia presents a significant challenge to public health systems. To effectively address this threat, we propose the following policy recommendations: (1) Develop and enforce standardized protocols for mosquito population monitoring and evaluation of vector control interventions. (2) Establish a regional network for real-time genomic sequencing and data sharing to monitor the evolution of CHIKV and guide evidence-based public health decisions. (3) Formulate national deployment strategies for CHIKV vaccines, prioritizing high-risk populations and implementing phased vaccination approaches based on epidemiological risk and resource availability. (4) Promote collaboration between China and ASEAN (Association of Southeast Asian Nations) countries to establish a coordinated surveillance and response framework for mosquito-borne diseases. (5) Strengthen foundational research to promote the advancement of drug development.
